# Human Type I Interferon Antiviral Effects in Respiratory and Reemerging Viral Infections

**DOI:** 10.1155/2020/1372494

**Published:** 2020-05-08

**Authors:** Patricio L. Acosta, Alana B. Byrne, Diego R. Hijano, Laura B. Talarico

**Affiliations:** ^1^Laboratorio de Investigaciones Infectológicas y Biología Molecular, Unidad de Infectología, Departamento de Medicina, Hospital de Niños Dr. Ricardo Gutiérrez, Buenos Aires (1425), Argentina; ^2^Consejo Nacional de Investigaciones Científicas y Técnicas (CONICET), Buenos Aires (1425), Argentina; ^3^Department of Infectious Diseases, St. Jude Children's Research Hospital, Memphis, TN 38105, USA

## Abstract

Type I interferons (IFN-I) are a group of related proteins that help regulate the activity of the immune system and play a key role in host defense against viral infections. Upon infection, the IFN-I are rapidly secreted and induce a wide range of effects that not only act upon innate immune cells but also modulate the adaptive immune system. While IFN-I and many IFN stimulated genes are well-known for their protective antiviral role, recent studies have associated them with potential pathogenic functions. In this review, we summarize the current knowledge regarding the complex effects of human IFN-I responses in respiratory as well as reemerging flavivirus infections of public health significance and the molecular mechanisms by which viral proteins antagonize the establishment of an antiviral host defense. Antiviral effects and immune modulation of IFN-stimulated genes is discussed in resisting and controlling pathogens. Understanding the mechanisms of these processes will be crucial in determining how viral replication can be effectively controlled and in developing safe and effective vaccines and novel therapeutic strategies.

## 1. Introduction

Type I interferons (IFN-I) are members from a large family of signaling proteins known for their potent antiviral activity. IFN-I were discovered in 1957 by Lindenmann and Isaacs and received their names based on the ability to interfere with influenza virus replication in chick cell culture [1–3]. In recent years, the knowledge about the mechanism of IFN-I production has quickly expanded.

IFN-I are produced by most cell types, the best known members of this group are IFN-*α* and IFN-*β*, and it also includes IFN-*ο*, IFN-*δ*, IFN-*κ*, IFN-*ε*, IFN-*τ*, and IFN-*ω* [4, 5], which directly mediate a potent antiviral response. IFN-*α* includes 13 partially homologous members, encoded by chromosome 9, while IFN-*β* is composed by a single member and the gene is located on chromosome 12 [4].

IFN-I production occurs primarily when pattern recognition receptors (PRRs) present on the cell surface or in the cytosolic compartment of virtually all cell types are stimulated by pathogen-associated molecular patterns (PAMPs) [6–8]. The most widely studied PRRs are Toll-like receptors (TLRs). Ten different TLRs have been identified in humans, all of which detect PAMPs either on the cell surface or the lumen of intracellular vesicles, such as endosomes or lysosomes, and are involved in the recognition of a particular type of PAMP [6]. TLRs recognize different pathogen components, including double-stranded RNA (dsRNA) (TLR-3), single-stranded RNA (ssRNA) (TLR-7), or CpG DNA [6, 9, 10]. Other PPRs include retinoic acid-inducible gene-I- (RIG-I-) like receptors (RLRs) and nucleotide-binding oligomerization domain- (NOD-) like receptors (NLRs).

**Figure 1:**
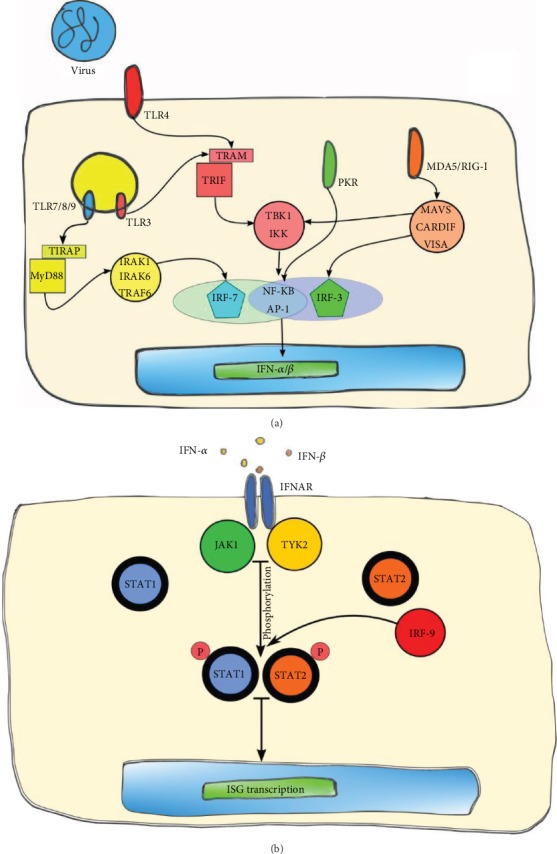
Type I interferon immune response. (a) Detection of viral infection by pathogen recognition receptors and signaling cascades resulting in the production of IFN-I. (b) IFN-I-*α*/*β* receptor (IFNAR) and activation of the JAK/STAT pathway leading to the induction of IFN-stimulated genes.

The recognition of TLR ligands leads to the recruitment of adaptor molecules that contain Toll interleukin-1 receptors (TIR) such as TIR-domain-containing adapter (TRIF), TRIF-related adaptor molecule (TRAM), Myeloid differentiation primary response gene 88 (MyD88), or TIR-domain-containing adaptor protein (TIRAP), leading the activation of molecular cascades that finally promote the activation of nuclear transcription factors such as nuclear factor *κ*B (NF-*κ*B), IFN regulatory factor 3 (IRF-3), and 7 (IRF-7) [11, 12], which leads to the induction of genes encoding IFN-I (Figure 1(a)).

Both IFN-*α* and IFN-*β* use the same receptor, IFN-*α*/*β* receptor (IFNAR), which is expressed on a vast variety of cell types [5] (Figure 1(b)). This receptor is associated with Janus kinase 1 (JAK1) and Tyrosine kinase 2 (TYK2). IFN-*α*/IFN-*β* binding to IFNAR activates JAK1 and TYK2, which subsequently phosphorylate the transcription factor signal transducer and activator of transcription 1 (STAT1) and STAT2 [13], among other less characterized transcription factors such as STAT3, STAT4, and STAT5. Once phosphorylated, these factors associate with IRF-9 to form the IFN-stimulated gene factor 3 complex (ISGF3). In the nucleus, this complex binds specific DNA sequences containing IFN-stimulated response elements (ISRE) that promote the transcription of hundreds of IFN-stimulated genes (ISGs) including IRF-1, IRF-7, IRF-8, and IRF-9, whose function is to inhibit viral replication and induce an antiviral response in the area of the infected cell [13–15]. Some ISGs have been widely studied; the best known members include IFN dsRNA-dependent protein kinase R (PKR), 2′-5′oligoadenilate synthetase (OAS), IFN-inducible transmembrane proteins (IFITM), dsRNA-specific adenosine deaminase (ADAR), and Myxovirus resistance protein A (MxA) and B (MxB) [16, 17].

The viral infectious cycle involves several steps, and ISGs target different stages of the viral cycle. For example, PKR, which was among the first ISG described, is a PRR that recognizes dsRNA, inducing IFN production and further ISG production [18]. PKR inactivates eIF-2*α*, leading to a global translation blockage of both viral and cellular mRNA [19]. OAS is also activated by cytoplasmic dsRNA and is subsequently involved in the degradation of RNA (through RNAse L), contributing to the inhibition of protein synthesis and therefore viral replication [20]. In the case of IFITM, the four members (IFITM-1, IFITM-2, IFITM-3, and IFITM-5) are present in endosomes and lysosomes and have a critical role in the inhibition of viruses that require vesicles for effective infection [21]. ADAR is a nucleic acid editing enzyme that disrupts base pairing. ADAR catalyzes the deamination of adenosine (A) to produce inosine (I) in dsRNA, inducing the replacement of the AU base pair by IU, which produces dsRNA destabilization [22]. On the other hand, MxA and MxB play a critical role during viral entry; while MxA acts in the early stages of infection once the virus has traversed the plasmatic membrane, MxB acts at the nuclear stage, inhibiting nuclear entrance [23, 24].

Dendritic cells (DCs) play a key role in the production of IFN-I. Although both myeloid DC (mDC) and plasmacytoid DC (pDC) produce IFN, the latter express specialized TLR-7 and TLR-9 and has a high constitutive expression of IRF-7 that allow them to respond to viruses with rapid and extremely robust IFN-*α* production [25–27]. Upon activation and production of IFN, pDCs mature into antigen-presenting cells (APC) serving as a key link between the innate and adaptive immune responses [28]. In addition, DCs produce IL-12, which drives naïve T cells into Th1-type responses, as well as IFN-*α*, which increases the frequency of IFN-*γ* producing CD4^+^ T cells [29]. Moreover, IFN-I also boost natural killer (NK) and CD8^+^ T cell responses, promoting both survival and clonal expansion of the latter [30–32]. IFN-*α* and IFN-*β* lead to an immediate innate antiviral response and stimulate the adaptive immune system, affecting myeloid cells, NK cells, T cells, and B cells to mount an adequate Th1-biased immune response [16, 33].

Since its discovery, IFN's role as an important player of the immune system against viral infections has become evident. Although IFN is known for its innate beneficial response, recent studies have also linked these immune mediators to pathogenesis.

This review summarizes the current knowledge of the roles of IFN-I in respiratory virus and reemerging flavivirus infections (Table 1) and on the strategies that different viruses adapt to subvert IFN-I responses, mainly focusing on studies in human cells and subjects.

## 2. Human IFN-I Responses to Viral Pathogens

### 2.1. Human Respiratory Viruses

Respiratory viruses including influenza virus, respiratory syncytial virus (RSV), human metapneumovirus (hMPV), parainfluenza virus (PIV), human rhinovirus (HRV), and human coronavirus (HCoV) are a major cause of respiratory disease and mortality in humans [34–36]. The disease can range in severity from mild or asymptomatic upper airway infections to severe wheezing, bronchiolitis, pneumonia, or death. Numerous factors can increase the risk of severe disease, including neurological conditions, chronic lung and/or heart disease, metabolic disorders, or a weakened immune system [37]. However, the majority of severe cases occur in previously healthy persons [34, 35].

When a cell is infected, the viral sensing activates pathways that trigger host immune responses. The viral presence is detected by several distinct PRRs including TLR-2, 3, 4, 6, 7, and 8, RLRs including RIG-I and MDA5 pathways among others [38–40]. These signaling pathways converge on IRF-3/IRF-7 and NF-*κ*B that promote the induction of IFN-I that inhibit viral replication and contribute to the initiation of more specific adaptive immune responses [40]. IFN-*α* and IFN-*β* bind to receptor complexes, which activate STAT1 and STAT2 phosphorylation. IRF-9 binds to STAT1/STAT2 heterodimers forming the ISGF3 complex, which translocates to the nucleus to induce transcription of ISGs and to mount an antiviral state within the cell [39, 41].

Respiratory viruses, like other pathogens, target core molecules of the immune cascade to evade the host response. Like other different viruses, one major strategy used by respiratory viruses is to modulate, evade, or inhibit the IFN response that allows viral replication and transmission [41–43].

#### 2.1.1. Influenza Virus

Influenza virus is a negative-sense, ssRNA virus that belongs to the *Orthomyxoviridae* family. This pathogen is a leading cause of respiratory illness in humans and causes annual epidemics and pandemics of differing severity [44]. The human subpopulations most vulnerable to influenza virus infections are children, pregnant women, and persons over 65 years old.

**Figure 2:**
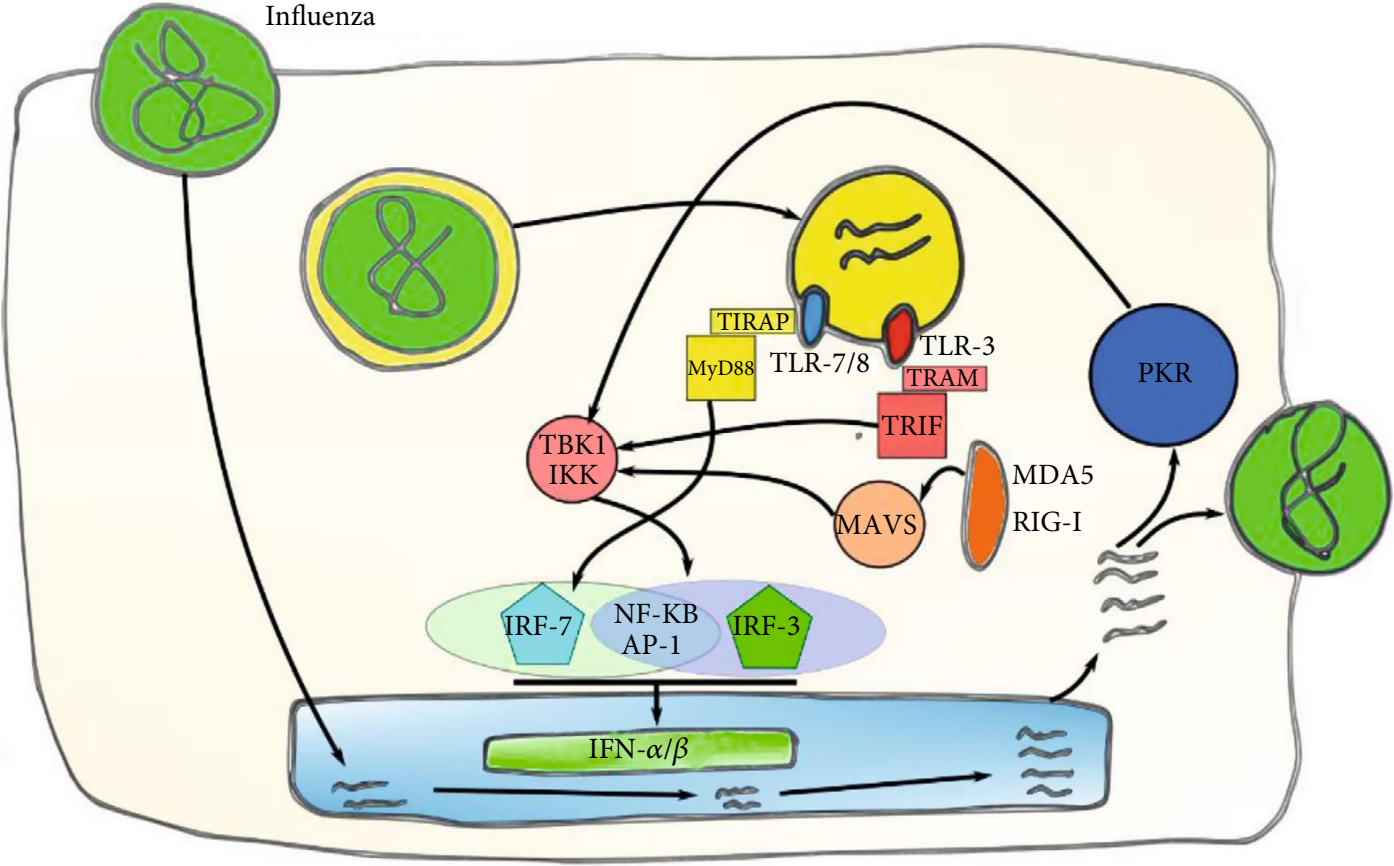
Induction of IFN-I by influenza virus. PRRs involved in viral recognition include TLR-3, TLR-7/8, MDA5, and RIG-I that can detect viral products to signal IFN-*α*/*β* production in infected cells.

Influenza virus is primarily recognized by two different types of PRRs: RLRs and TLRs. Regarding the RLR family, RIG-I, LGP2, and MDA5 sense viral RNA during viral replication in the cytoplasm [45–47]. Once activated, RIG-I and MDA5 interact with the mitochondrial antiviral-signaling protein (MAVS), leading the activation of NF-*κ*B and IRF-3 to stimulate IFN production (Figure 2). On the other hand, LGP2 has been related to the regulation of RIG and MDA5 activation [48]. Two different TLRs are activated by influenza virus, TLR-3 and TLR-7. The stimulation of TLR-3 in DCs and macrophages signal through TRIF, activating IRF-3/ NF-*κ*B, leading to IFN-*β* secretion. On the other hand, TLR-7 in DCs activates MyD88, which activates IRF-7 and produces the secretion of both IFN-*α* and IFN-*β* [49, 50].

**Figure 3:**
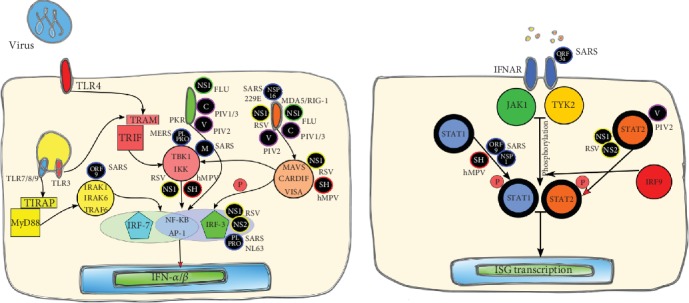
Evasion and inhibition of respiratory virus-induced IFN-I signaling. Many respiratory viruses can inhibit and modulate their detection using diverse strategies, thereby inhibiting the IFN-I production. Circular boxes in black background represent the viral protein involved in the pathway inhibition. Abbreviations: NS: nonstructural protein; V: V protein; C: C protein; SH: small hydrophobic protein.

Influenza virus NS1 suppresses IFN synthesis through several different mechanisms [51–54] (Figure 3). First, it limits the pretranscriptional induction of IFN by forming a complex with RIG-I and limiting its signaling [55, 56]. Second, it directly limits the extent of the antiviral state by inhibiting PKR by sequestering dsRNA and by forming a complex with PKR avoiding its activation [57, 58]. Third, it inhibits mRNA maturation by interfering with the effector domain of the 3′-end processing [59]. Finally, NS1 activates phosphatidyl-inositol-3 kinases (PI3K) which can delay apoptosis of infected cells [60].

The evidence supporting the role of IFN-I during influenza infection in human populations is limited. Much of the available information focused on animal studies that have yielded mixed results. While some studies have found a protective role of IFN-I, others found no effect or even a pathogenic role [61–64]. Originally, Hall et al. detected that increased IFN production in nasal washes in children infected with influenza virus was associated with decreased shedding of virus [65]. Subsequent studies identified the high levels of IFN-I in breastfeeding children infected with influenza virus, providing support to the hypothesis that breastfeeding protects against respiratory viral infection [66, 67]. Additionally, a study of elderly subjects demonstrated that IFN-*α* production is decreased during influenza virus infection and suggested that this impairment can produce multiple defects in their innate and adaptive immune responses that could lead to increased severity [68]. Likewise, other studies have shown that pregnant women have an attenuated IFN-*α* response to influenza virus [69].

As mentioned previously, IFITM are a family of interferon-induced antiviral restriction factors with constitutive expressions in different cell types that are known to be induced by IFN-I. Recent evidence has shown that ISG IFITM3 is a potent antiviral factor in restricting influenza virus infection and a decrease in its expression results in a higher risk of hospitalization [70, 71].

#### 2.1.2. Respiratory Syncytial Virus (RSV)

RSV is a common virus that belongs to the *Paramyxoviridae* family. RSV is a negative-sense ssRNA virus that causes a wide range of symptoms and the severity can vary from mild to lethal disease [59]. RSV is especially severe in infants, the elderly, and immunocompromised individuals [72, 73]. Despite years of research, RSV is the only major etiological agent of acute lower respiratory tract infection (ALRI) for which no vaccine or specific treatments are available.

**Figure 4:**
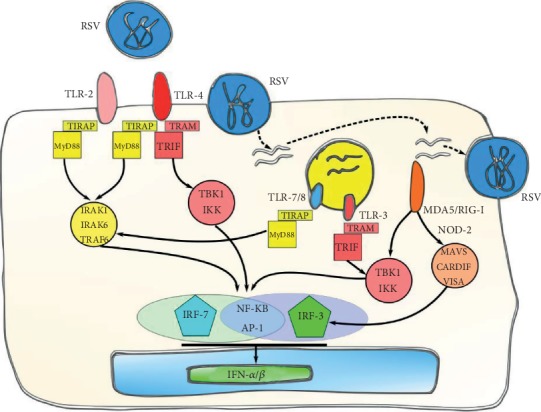
Induction of IFN-I by RSV. RSV is detected by different PRRs that include TLR-2, TLR-4, TLR-3, TLR-7/8, MDA5, and RIG-I and leads to the IFN-I pathway activation.

Several PRRs are involved in RSV recognition, including TLRs (TLR-2, TLR-3, TLR-4, and TLR-7), RLRs (RIG-I and MDA5), and NLRs (NOD-2). IFN-I production during RSV infection is activated by different mechanisms (Figure 4). TLR-3 detects dsRNA during viral replication and signals through TRIF, which activates IFN-*α* production through NF-*κ*B and IRF-3 pathway [74]. Other important PRRs for IFN-I production are RIG-I and MDA5 which subsequently link MAVS. This recruits TRAF2/6 and TRAF3 which leads to the activation of IRF-7 and IRF-3 through NF-*κ*B [75]. Together, these IRFs stimulate ISRE sequences that promote IFN-I production [76, 77]. NLRs also play a role in activating the IFN-I pathway. NOD-2 can recognize RSV ssRNA, thus, mediating IRF-3 and NF-*κ*B activation pathways and leading to the IFN-*β* production [78].

Although RSV can lead to IFN-I production through multiple pathways, it has been established that this virus in particular is a poor stimulator of IFN-I. Unlike other respiratory viruses, studies have shown a limited role for IFNs in response to RSV infection [65, 79, 80]. Thereby, NS1 and NS2 (2 proteins encoded by RSV) appear to interact (and interfere) with multiple molecules of the IFN pathway (RIG-I, TRAF3, and IRF-3), which then interfere with IFN synthesis [81–84] (Figure 3). Studies of RSV NS1 and NS2 proteins have demonstrated that they are crucial for virus replication *in vitro*, however, through IFN-I antagonism, they contribute to adequate RSV replication *in vivo* (and *in vitro*) [85]. Using interference RNA that allowed the inhibition of NS1, the expression of IFN-*α* and IFN-*β* was augmented and this increase was associated with a reduction in viral titer [86]. On the other hand, NS2 has been shown to be the main IFN-I antagonist related to the STAT-2 pathway [82, 87], thus, inhibiting IFN-*α* and IFN-*β* responses through JAK/STAT signaling [82, 88].

One interesting study by Marr et al. showed that RSV-induced IFN-*α* expression by primary pDCs exposed to RSV is strongly correlated with age, observing higher IFN-*α* expression in healthy adults, followed by preschool children and little to no expression in healthy full-term infants [89]. This analysis of developmental innate immunity associated with poor IFN-I production during the first year of life suggests a role for IFN-I in RSV pathogenesis. Deficits in MAVS or RIG-I signaling events could explain this deficit. Furthermore, infants with severe bronchiolitis have less IFN-I levels when compared to those with moderate disease [90]. Taken together, these results suggest a critical role for IFN-I in RSV infection.

#### 2.1.3. Human Metapneumovirus (hMPV)

hMPV is a member of the *Paramyxoviridae* family, genus *Metapneumovirus* [91]. Isolated in 2001 [91], hMPV is a nonsegmented negative-strand RNA virus [92] and has been associated with the upper and lower respiratory tract infections with symptoms ranging from colds to pneumonia. hMPV particularly affects children, the elderly, and immunocompromised individuals [93]. Worldwide, hMPV is recognized as the second most common cause of bronchiolitis and pneumonia in children under 5 years old [94]. Despite the fact that hMPV is a clinically relevant pathogen, no vaccine is currently available.

Similar to RSV, two major pathways for the secretion of IFN-I during hMPV infection have been described. Each pathway involves different types of PRRs, one utilizes RLRs and the other TLRs. The former is primarily activated by RIG-I and MDA5, whereas the latter involves TLR-3 and TLR-7. RIG-I- and MDA5-mediated signal transduction begins when viral RNA is sensed in the cytosol. This recognition leads to the activation of IRF-3 and IRF-7 through the MAVS pathway [75, 95, 96]. TLR-3 activates the IFN pathway through IRF-3 and TLR-7 via IRF-7.

It is worth noting that the vast majority of cells express IRF-7 only after the activation of IRF-3 (which is expressed constitutively and is activated essentially by the RIG-I/MAVS pathway) or in response to IFNs. Importantly, TLR-7 is the main PRR that participates in sensing hMPV by pDCs, whereas MDA5-MAVS is the major pathway mediating hMPV sensing in conventional DCs [75].

Similar to other respiratory viruses, evidence suggests that hMPV has mechanisms for evading IFN-I production through interference in TLR- and RLR-dependent surveillance pathways. Early studies published by Dinwiddie et al. showed that hMPV can inhibit the IFN-*α* pathway in A549 cells. Therefore, this virus abolished the IFN-*α* pathway and downstream ISG signaling through mechanisms that regulate STAT1 activation [97]. One study supporting this information found that hMPV suppressed IFN-I responses through mechanisms involving the regulation of STAT1, STAT2, JAK1, TYK2, and the surface expression of IFNAR1 [98]. hMPV proteins impair the activation of PPRs through several different mechanisms. hMPV M2-2 protein interferes with MyD88 adaptor, a critical component for the activation of proinflammatory genes. This protein also interacts with MAVS, altering the production of IFN-*β* and also prevents IRF-3 phosphorylation [99]. In addition, hMPV G protein alters the recognition by RIG-I thereby influencing the secretion of IFNs, and the small hydrophobic protein can inhibit NF-*κ*B, which is an important component in the IFN pathway [100]. Finally, the hMPV phosphoprotein B1 interferes with RIG-I recognition [75].

Studies describing the role of IFN-I in hMPV infections in humans are very limited. One study has shown that IFN-*β* was induced after hMPV infection in children [101]. In addition, a deleterious effect of IFN-I was reported in a murine model, in which IFN-I contributed to disease pathogenesis due to increased inflammatory lung disease during infection [102].

#### 2.1.4. Parainfluenza Viruses (PIVs)

PIVs are a group of nonenveloped, negative-sense, ssRNA viruses that belong to the *Paramyxoviridae* family. First isolated in the 1950s, PIVs are composed of five different (antigenic and genetic) types, PIV-1, PIV-2, PIV-3, and PIV-4 with two subtypes PIV-4a and PIV-4b [59, 103]. The virus was named *Parainfluenza* because it produces influenza-like disease and has a lipid envelope and hemagglutination and neuraminidase activities [59]. Worldwide, PIVs are important causes of upper and lower respiratory tract illnesses. Although PIV infections are generally self-limiting, some patients require hospitalization and the disease may lead to mortality, especially in children under the age of five years [104], the elderly, and the immunocompromised individuals.

All PIVs encode 6 universal proteins, N, P, M, F, HM, and L, and at least one additional protein from the P gene (C, V, D, W, and I), which is not essential for viral replication [59].

The innate immune response to PIV is not well-characterized, and little is known about the signaling pathways. Unlike other respiratory viruses, TLR signaling is not described but is thought to play a role in the IFN pathway activation during PIV infection. Regarding RLRs, a study showed that RIG-I is involved in IFN-I induction via IRF-3 after PIV-3 infection [105]. In addition, MDA5 has also been found to be activated by degraded products of RNase L from PIV [106].

This group of viruses is known to encode proteins that block innate immune responses to viral infections, allowing PIV replication. As mentioned above, pathogen RNA synthesis provides strong stimuli to mount an IFN immune response. PIV-1 and PIV-3 encode C proteins, while PIV-2 encodes a V protein. Both proteins, C and V, are involved in the blockade of IFN-I induction by preventing PKR activation [107–110]. The viral strategy in the case of PIV-1 and PIV-3 is to avoid the IFN production. PIV-1 does not inhibit the IFN pathway, given that the synthesis of the viral RNA can activate IFN production. Rather, the viral C protein modulates its RNA production, preventing MDA5 activation [109, 111]. This protein also interferes with STAT1, avoiding signaling through this pathway and thus IFN production [112]. The V protein of PIV-2 interferes with the IFN production in different ways. One of the mechanisms is the inhibition of MDA5 activation [113]. In addition, the highly conserved V protein Cys-rich domain has been shown to be both necessary and sufficient to limit the activation of the IFN-*β* promoter [114]. Finally, the V protein interferes with the IFN pathway by abolishing STAT2 signaling [115]. Interestingly, there is evidence that shows that this protein also regulates viral RNA production (as PIV C protein) [116].

Overall, little is known about IFN-I response after the PIV infection in human population studies. An early work detected IFN-I in patients with primary PIV infection [65]. *In vitro* experiments have shown that PIV-2 induced IFN-*α* by day 2 postinfection, PIV-3 by day 3, and that PIV-1 did not produce this molecule. IFN-*β* production was shown to be poor in all serotypes [117].

#### 2.1.5. Human Rhinovirus (HRV)

HRV is a small, nonenveloped, positive-sense, ssRNA virus that belongs to the *Picornaviridae* family. The family is divided into three species: rhinovirus A, B, and C [59]. HRV is a ubiquitous seasonal microorganism and is the most frequent cause of common cold (causing more than 50% of upper respiratory tract infections in humans worldwide). Given that the viral genetic diversity is huge (>160 serotypes), recurrent infections with this virus are frequent. Although HRV infections are not life-threatening, they can be also detected in the lower airways where they can cause severe exacerbations in patients with asthma and chronic obstructive pulmonary disease [118, 119].

Different PRRs are involved in HRV recognition, belonging to two different families of receptors: TLRs (TLR-2, 3, 7, and 8) and RLRs (RIG-I and MDA5). TLR-2 on the cell surface recognizes the virus capsid, even without viral replication. In addition, TLR-3, TLR-7, and TLR-8 localized in intracellular compartments are stimulated once the viral particle is internalized and the dsRNA/ssRNA is sensed [120]. The TLR stimulation leads to the activation of downstream signaling molecules that activate IRF-3, IRF-7, and NF-*κ*B, triggering IFN-I secretion. The evidence regarding TLR-3 is somewhat unclear; while one study described an important role for this receptor in the host response against the HRV infection [121], others found no function for this PRR [120]. The HRV genome is also recognized by RIG-I and MDA5 (both localized in the cytosol) which can recognize ssRNA and dsRNA, respectively. The RLR stimulation leads to the activation of MAVS and the consequent activation of NF-*κ*B and IRF, triggering the production of IFN-I in the airway cells [120, 122, 123]. Responses mediated by IFN-I *in vivo* are critical for the antiviral effects that limit HRV through the activation of NK cells [124].

Similar to other respiratory viruses, HRV seems to suppress IFN synthesis using multiple strategies. Even though IFN evasion mechanisms have been well-studied in other enteroviruses, the information for HRV is scarce. A study showed that the MAVS adaptor is cleaved by 2A and 3C proteases of HRV to stop IFN-I signal transduction [125]. Furthermore, the 3C proteases have been implicated in the cleavage of RIG-I altering IFN-I production [126]. Regarding TLRs, despite their crucial role in viral sensing, there is no evidence of TLR signaling evasion by HRV.

Different studies have attributed susceptibility to HRV infection to impaired IFN-I production that leads to a higher viral load [127, 128]. Abnormal HRV-induced IFN in asthmatic patients was shown in peripheral blood mononuclear cells (PBMCs) for IFN-*α* and in primary human bronchial epithelial cells for IFN-*β* [129]. In addition, *in vitro* studies on epithelial cells showed that exogenous IFN-I led to reduced viral load [130]. Furthermore, this has been confirmed in epithelial cells from asthmatic patients, suggesting a role for IFN-I in limiting induced viral exacerbations [131]. However, other groups have found no differences between viral loads in asthmatic and control subjects during HRV infections [132]. Further investigation regarding the role of IFN-I during HRV infections needs to be completed.

#### 2.1.6. Human Coronavirus (HCoV)

The family *Coronaviridae* contains two subfamilies, the *Coronavirinae* and the *Torovirinae*. They are a large group of positive-sense ssRNA genome viruses that can infect mammals and birds, causing a wide variety of diseases which can lead to frequent mutations and infections of new species. They have been divided into four genera, two of which, alphacoronavirus and betacoronavirus contain viruses infecting humans [133]. To date, four HCoVs (HCoV-229E, HCoV-NL63, HCoV-OC43, and HCoV-HKU1) circulate globally in the human population and contribute to approximately one-third of common cold infections in humans [134]. HCoVs had been regarded as mildly pathogenic until the early 2000s. At that time, a new disease appeared in China, severe acute respiratory syndrome (SARS), which was quickly attributed to a new HCoV, the SARS-CoV. Following that outbreak, a related but different HCoV producing severe respiratory disease emerged, the Middle East respiratory syndrome coronavirus (MERS-CoV) [135]. Recently, a novel coronavirus (2019-nCoV) has emerged. The 2019-nCoV is causing an outbreak of unusual viral pneumonia in patients. It initially began in Wuhan, China and has spread to many countries worldwide [136].

Similar to previously described respiratory viruses, HCoVs are detected by TLR-3, TLR-7, RIG-I, and MDA5 [137]. TLR-3 activated by dsRNA stimulates TRIF that activates AP-1, NF-*κ*B, IRF-3, and IRF-7 leading to the IFN production [138]. TLR-7 senses the ssRNA, leading to the activation of IRF-7 that also stimulates IFN secretion. RIG-I and MDA5 sense viral molecules in the cytoplasm, and their activation stimulates MAVS that induces the activation of IRF-3 and NF-*κ*B to finally induce the IFN production [139].

Like other viruses, HCoVs use multiple mechanisms to evade IFN-I immune response. Evidence shows that different viral structures can inhibit or modulate the IFN production or activity. Thereby, NS protein-16 codified by SARS-CoV and HCoV-229E induces the methylation of viral mRNA cap structures that inhibit recognition by MDA5 [140, 141]. The papain-like protease (PLPRO) domain of NS protein-3 of SARS-CoV and HCoV-NL63 inhibits the activation of IRF-3 [142, 143]. Moreover, it was demonstrated that PLPRO encoded by MERS-CoV suppresses the induction of IFN-*β* through its deubiquitinating activity [144]. Protein M of SARS-CoV inhibits IFN-I production by impeding the formation of the TRAF3-TANK-TBK1/IKK*ϵ* Complex [145]. ORF9b protein of SARS-CoV was shown to stimulate the degradation of MAVS and TRAF6, inhibiting the IFN-I pathway [146].

The IFN response seems to be contributing simultaneously to the protection against viruses and to the pathology induced by the same viral infections. Human pDCs demonstrate a robust IFN-I production after MERS-CoV infection, especially IFN-*α*, and this response is greater than that elicited by SARS-CoV [147]. High IFN-*α* and IFN-*γ* have been associated with early SARS-CoV sequelae suggesting that unregulated IFN responses during acute-phase SARS-CoV may be deleterious for this infection [148].

### 2.2. Reemerging Flaviviruses

The interplay of climatic and environmental changes as well as the growth of the human population and increased urbanization has triggered the reemergence and rapid spread of arthropod-borne viruses significant to public health. Yellow fever virus (YFV), dengue virus (DENV), West Nile virus (WNV), and Zika virus (ZIKV) are mosquito-borne flaviviruses that have reemerged in both hemispheres during recent decades. Other flaviviruses have emerged in specific regions of the world, including the Japanese Encephalitis virus (JEV) and Saint Louis encephalitis virus (SLEV), among others [149].

DENV, ZIKV, WNV, YFV, JEV, and SLEV belong to the flavivirus genus of the *Flaviviridae* family, which comprises a diverse group of enveloped, positive-sense, ssRNA viruses transmitted by blood-feeding mosquitoes, causing disease in humans [150]. The flavivirus RNA genome (11 kb), which encodes a single open reading frame flanked by highly structured 5′ and 3′ untranslated regions (UTRs), is transcribed as a single polyprotein that is proteolytically processed by host and viral proteases to yield three structural proteins (C, prM, and E) and seven nonstructural proteins (NS1, NS2A, NS2B, NS3, NS4A, NS4B, and NS5), the latter of which regulate viral translation, transcription, and replication as well as attenuate host antiviral responses [150].

**Figure 5:**
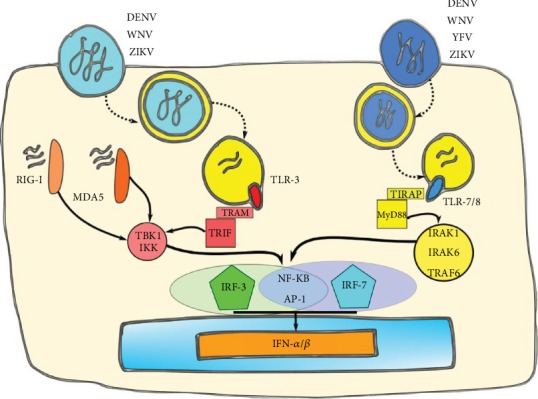
Induction of IFN-I by flaviviruses. The TLR and RLR signaling cascades converge with the activation of transcription factors, which are critical for the induction of IFN-*α*∕*β*.

In human cells, the host responds to flavivirus infection by recognizing viral nucleic acids through several distinct PRRs including RLRs, TLR-3, 7, and 8, NLRs, and the cyclic GMP-AMP synthase/stimulator of IFN genes- (cGAS-STING-) dependent sensing pathway [151, 152] (Figure 5). Among RLRs, RIG-I and MDA5 are involved in the detection of cytoplasmic dsRNA produced during viral replication [153]. The TLRs of importance during flavivirus infections are TLR-7 and TLR-8, which detect ssRNA as well as TLR-3, which identifies dsRNA produced during viral replication [152, 153]. Recently, it has been reported that DENV infection activates TLR-9 signaling, which is known to recognize bacterial or viral DNA, by inducing mitochondrial DNA (mtDNA) release in human DCs [154]. The cGAS-STING pathway, which is known to sense DNA viruses, has also been recently involved in restricting flavivirus infections [151]. It has been reported that during DENV infection, mtDNA is spilled into the cytoplasm and this subsequently activates the cGAS/STING signaling pathway to stimulate the production of IFN-I [155]. pDCs are a predominant source of IFN-I during viral infection and TLR7 signaling in pDCs has been reported to promote the contact of these cells with infected cells in a specialized platform that enables viral RNA transfer and antiviral responses [156]. The binding of viral ssRNA and/or dsRNA to PRRs activates downstream signaling cascades, such as the activation of transcription factors IRF-3 and IRF-7 and NF-*κ*B, that result in the induction of IFN-*α* and -*β*. Subsequent secretion of IFN-I and binding to IFNAR activates JAK/STAT dependent- and independent-signaling cascades that result in the transcription of hundreds of ISGs, which encode proteins that inhibit flavivirus replication and spread [151, 153, 157].

**Figure 6:**
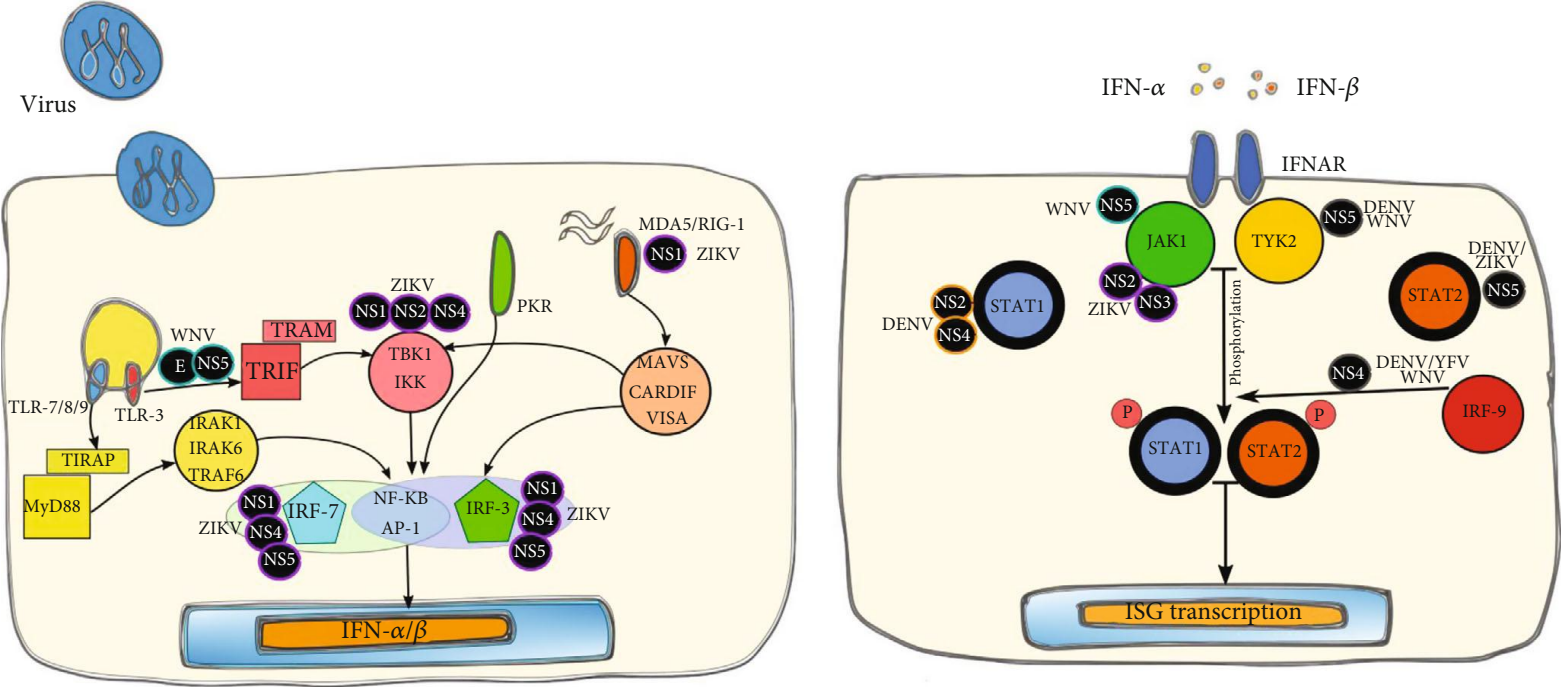
Inhibition of the IFN-I pathway by flaviviruses. Different viral proteins are involved in the modulation of the IFN-I activation pathway. Circular boxes in black background represent the viral protein involved in the pathway inhibition. Abbreviations: nonstructural protein (NS) 1, 2, 3, 4, and 5; E: envelope protein.

To facilitate propagation, flaviviruses, like other viruses, have evolved specific strategies involving one or more viral nonstructural proteins to either prevent IFN induction or to inhibit IFN signaling. The inhibition of IFN-I induction is achieved by either sequestration or modification of viral RNA and inhibition of PRRs [151]. In addition, several flavivirus nonstructural proteins such as NS2A, NS2B-NS3, NS4B, and NS5 have been shown to interfere with IFN signaling pathways through different mechanisms depending on the virus [152, 158, 159] (Figure 6). Recently, subgenomic flavivirus RNA (sfRNA), a nongene product encoded in 3′UTR generated by incomplete degradation of viral RNA by a cellular 5′-3′ exoribonuclease, has been proposed to play a modulatory role in the host antiviral response in mammalian cells by antagonizing IFN-I, as well as displaying viral interference in insect cells [24, 160–162].

The protective role of IFN-I against flaviviruses has been extensively demonstrated in mice since IFN-I signaling-deficient mice are highly susceptible to infection by DENV, ZIKV, and WNV [163–166]. The reported effects of IFN-I in flavivirus infections in human cells and subjects are discussed in the following sections.

#### 2.2.1. Dengue Virus (DENV)

DENV is an acute febrile disease caused by four distinct antigenically related DENV serotypes (DENV-1, -2, -3, and -4) that are transmitted to humans by the bite of *Aedes* spp. mosquitoes, mainly *Aedes aegypti* [167]. DENV infects an estimated 390 million people every year of which 96 million have apparent DENV infections, with different levels of disease severity [168]. The clinical manifestations of dengue can range from mild febrile illness with myalgia and rash (formerly known as dengue fever (DF)) to severe forms of disease, characterized by plasma leakage and hemorrhage (dengue hemorrhagic fever (DHF)) leading to potentially life-threatening hypovolemic shock [169]. At present, several DENV vaccines are under development, including one that has been registered in several countries with high endemicity due to the limited efficacy in naïve individuals and against all four DENV serotypes [170].

DENV has been reported to trigger a robust IFN-I response, which has been shown to be important in controlling DENV infection [171]. IFN-I has been reported to inhibit DENV infection in a variety of human cells, including hepatoma, fibroblasts, and myeloid cells [172, 173]. RIG-I and MDA5 sensing of DENV has been critical in the immune response [39], as knockdown of RIG-I and MDA5 in Huh7 cells resulted in increased DENV replication [174]. In addition to TLR-3, 7, and 8 [151], DENV has been reported to activate and upregulate the expression of TLR-2 and 6 in human PBMCs [175]. NLRs have also been shown to be activated in DENV and WNV infections leading to the formation of the inflammasome complex with subsequent production of inflammatory cytokines of the IL-1*β* family [176]. DENV sensing by PRRs results in the secretion of IFN-I which triggers the JAK/STAT pathway leading to the production of ISGs with diverse antiviral properties [151].

DENV nonstructural proteins have been involved in the downregulation of the IFN pathway in humans, targeting important signaling molecules downstream of PRRs, resulting in the inhibition of IFN-regulated gene expression [177]. NS4B in combination with NS2A and NS4A have been reported to block IFN-I signaling by decreasing STAT1 phosphorylation in human A549 cells [178]. Furthermore, the DENV NS2B-NS3 protease complex has been involved in the cleavage of the human adaptor molecule STING or MITA, inhibiting IFN-I production [179, 180]. Interestingly, DENV cleaves STING in humans but not in nonhuman primates that may serve as its sustaining reservoir in nature [181]. In addition, the DENV NS2B-NS3 protease interacts with the cellular I*κ*B kinase, an important kinase involved in the IFN-I induction, disrupting RIG-I signaling and inhibiting the IFN production [182]. The DENV polymerase NS5 has also been reported as a potent and specific IFN-I antagonist, due to its binding to human STAT2 for ubiquitin-mediated proteasomal degradation [183, 184]. The ability of DENV NS5 to bind and degrade human but not mouse STAT2 may be the major reason behind DENV's efficient replication in human but not in wild-type mouse cells [185]. Recently, increased virulence of different type 1 DENV isolates has been associated with a higher capacity of NS proteins to suppress IFN signaling [186]. Likewise, sfRNA of DENV strains that are associated with greater epidemic potential prevents the ubiquitination-dependent activation of RIG-I by binding to the ubiquitin ligase tripartite motif protein 25 (TRIM25), subverting the RIG-I pathway, and consequently impairing IFN-I induction [162].

The viral control and immune regulation exerted by IFN-I in dengue patients and human cells infected with DENV have been studied in several reports [172, 173, 187–189]. Even though a strong IFN-I response has been described in dengue patients, the association of this response with disease severity is controversial [188, 190–192]. While some studies reported similar serum levels of IFN-*α* or IFN-*β* in DF and DHF in Thai and Mexican patients [188, 191], several other reports from Brazil, Mexico, Colombia, Taiwan, India, and Thailand showed higher levels of IFN-*α* in patients with milder dengue disease [77, 190, 193–196], suggesting that a robust production of IFN-*α* may be correlated with a better clinical condition with respect to dengue infection and disease progression. Conversely, a recent study from Paraguay reported higher levels of IFN-*β* in severe *vs.* nonsevere dengue children [197]. Similarly, a study from Northeast Brazil described higher levels of IFN-*β* in primary DHF patients compared to those with primary DF [198]. Considering the preexisting immunity of patients to DENV, several reports described higher levels of IFN-*α* in patients undergoing primary infection compared to those with secondary DENV infections [77, 190, 192, 195], while other studies observed a similar expression of IFN-*α* levels in both types of infections [197, 198]. Furthermore, different serum levels of IFN-*α* and IFN-*β* have been reported in DENV-infected patients [192], probably due to the differential kinetics of IFN-I induction during DENV infection of human cells [199, 200].

#### 2.2.2. Zika Virus (ZIKV)

ZIKV is a newly emerging flavivirus transmitted to humans by *Aedes* spp. mosquitoes, including *Aedes aegypti* [201]. Since the first human ZIKV infection was reported in Uganda in 1964 [202], human ZIKV infections had remained sporadic and limited to small-scale epidemics in Africa and Southeast Asia for decades [203, 204], until 2007, when a large outbreak of Zika fever was reported on Yap Island (Micronesia) [205]. Over the next seven years, outbreaks were reported in other Pacific Islands [206]. In 2015, the largest outbreak of ZIKV ever reported began in Brazil, with a rapid expansion of the virus in South and Central America [207–209]. Typically, ZIKV infections can be asymptomatic or manifested as a self-limiting febrile illness characterized by rash, headache, conjunctivitis, arthralgia, and myalgia [204–207, 209]. However, recent outbreaks in the South Pacific and Latin America reported severe neurological complications, including Guillain-Barré syndrome (GBS) in adults and microcephaly in newborns [210–212]. ZIKV has been shown to cross the placental barrier, disrupting brain development [213]. Recently, ZIKV spread by sexual contact has also been documented [214]. At present, there is an urgent need for vaccines and therapeutics to combat ZIKV.

The induction of antiviral immune responses after ZIKV infection of human cells has been reported in several studies [215–219]. The innate immune response after ZIKV infection of skin cells and endometrial stromal cells was characterized by a strongly enhanced IFN-*β* gene expression and the induction of ISGs, including OAS, ISG15, and MX1 [215, 216]. Moreover, the transcription of PRRs such as TLR-3, RIG-I, and MDA5 has been reported to be induced upon ZIKV infection of human skin cells [215]. Similar to DENV, NLR (inflammasome) activation has also been reported in ZIKV infection of monocytes [220]. Elevated secretion of IFN-*β* was also reported in human lung epithelial cells, which may delay the apoptosis exerted by ZIKV infection [217]. Schwann cells (SC), which play a central role in peripheral nerve disease and can be the target for damage in GBS, were susceptible to infection with ZIKV and YFV, but not DENV, and ZIKV infection of SC induced expression of IL-6, IFN-*β*, IFN-*λ*, IFIT-1, TNF-*α* and IL-23A mRNAs, and negative regulators of IFN signaling [218]. Furthermore, ZIKV infection of human DCs produced a strong induction of the RLR signaling pathway at the RNA transcription level, but ZIKV was able to block IFN-I signaling by targeting STAT1 and STAT2 phosphorylation [219].

The mechanisms by which ZIKV antagonizes IFN-I induction and signaling in human cells, which might contribute to the broad cellular tropism and persistence of ZIKV, have been reported in recent studies [159, 221–223]. ZIKV NS1 has been reported to interact with RIG-I and downregulate the antiviral signaling pathway [224]. In addition, Donald et al. reported that sfRNA in ZIKV acts as the antagonist of RIG-I-dependent IFN production [161]. Furthermore, ZIKV NS4A impairs RLR-MAVS interaction and subsequent induction of antiviral immune responses by binding to the caspase activation and recruitment domain (CARD) of MAVS and thus blocking its accessibility by RLRs [225, 226]. ZIKV NS3 has been shown to antagonize antiviral gene induction by RIG-I and MDA-5 by binding to and sequestering the scaffold proteins 14-3-3*ε*/*η* [227]. ZIKV NS1, NS2A, NS2B, and NS4B have been shown to interact directly with TANK-binding kinase 1 (TBK1), required for the phosphorylation of IRF-3 [159, 228]. In addition, the NS2B-NS3 protease complex of ZIKV has been reported to impair the JAK/STAT signaling pathway by promoting the degradation of JAK1 and to block RLR-triggered apoptotic cell death. Furthermore, the cooperation between NS1, NS4B, and NS2B-NS3 further attenuates antiviral immunity, by impairing IFN-induced degradation of NS2B-NS3 [159]. Similar to DENV, ZIKV NS5 has been reported as a potent antagonist of IFN-I responses by targeting human but not mouse STAT2 for ubiquitin-mediated proteasomal degradation [183, 185, 221, 222]. However, unlike DENV, ZIKV NS5 did not require the E3 ubiquitin ligase UBR4 interaction, exhibiting a virus-specific mechanism [222]. In addition, ZIKV NS1 and NS5 interact with NLRP3 and promote assembly of the NLRP3 inflammasome complex resulting in IL-1*β* production and stimulated ZIKV replication [229–231].

Recently, ZIKV microcephaly in newborns has been associated with rs3775291 single nucleotide polymorphisms (SNP) at TLR-3 reducing the activation of NF-*κ*B and thus decreasing IFN-I responses in mothers infected by ZIKV during pregnancy [232]. Furthermore, comparative analysis of African and Asian lineage-derived ZIKV strains revealed pronounced differences in the activation of innate immune signaling and inhibition of viral replication which may be related to differential pathogenesis [233]. Compared to the replication of other ZIKV strains, the replication of Asian ZIKV strain Brazil Fortaleza 2015, which was associated with neurodevelopmental disorders, was less sensitive to the antiviral actions of IFN-I, while infection with this strain induced weaker and delayed innate immune responses *in vitro*.

#### 2.2.3. West Nile Virus (WNV)

WNV is a mosquito-borne flavivirus of international health concern. WNV is maintained in a natural cycle involving primarily *Culex* spp. mosquitoes and avian hosts, but it also infects humans, horses, and other animals [234]. WNV is endemic in parts of Asia, Africa, and Europe, and it was first introduced to North America in 1999 [235]. Since then, the virus has spread rapidly throughout the United States and has been associated with over 21,000 encephalitis/meningitis cases and 1,800 deaths [236]. However, no effective prophylactic or therapeutic measures are currently available [234]. Infection with WNV in humans remains asymptomatic and/or subclinical in most cases and causes symptoms in 20-30% of WNV-infected individuals. The clinical manifestations range from a mild flu-like illness to more severe neuroinvasive disease, associated with significant morbidity and mortality [234, 237].

The protective role of IFN-I after WNV infection has been extensively demonstrated in mice by the marked increase in mortality in infected IFN-*α*/*β* receptor-deficient mice [166]. The studies of IFN-I protection after WNV infection are more limited in human cells. Recently, the PI3K, which play an important role in the induction of IFN-I antiviral responses, have been shown to control West Nile virus infection in human and mouse cells [238]. The presence of PI3K inhibitors blocked the translocation of IRF-7 from the cytosol to the nuclei, reduced IFN-I mRNA and protein expression, and decreased the secretion of IFN-I. Recently, in human monocyte-derived DCs early activation of RLR or IFN-I signaling could block WNV infection [239]. Furthermore, in human glioblastoma cells, WNV replication was regulated by early IFN-*β* induction, while in human neuroblastoma cells, a delayed IFN-*β* response, due to the concealing of viral dsRNA in intracellular membranes, resulted in efficient WNV replication [240]. In addition, differences in replication and induction of IFN-I responses between attenuated and virulent WNV strains in human monocyte-derived DCs accounted for the differing virulence in humans [241].

WNV NS4B and NS5 have been reported as important IFN-I antagonists [178, 242]. Similar to DENV, NS4B inhibited the JAK/STAT signaling pathway by decreasing STAT1 phosphorylation [178]. The NS5 of WNV strain NY99 blocked IFN-I responses by inhibition of STAT1 phosphorylation [242]. The helicase domain of WNV NS3 has been reported to inhibit IFN-I signaling and differences in virulence between the NY99 and NSW2011 strains of WNV have been attributed to differential inhibition of the helicase domain of NS3 [243]. Furthermore, WNV NS1 has been demonstrated to antagonize IFN-*β* production by suppressing RLR activation [244].

The human studies evaluating the IFN-I response to WNV infection and its relationship with disease severity are scarce [245]. Recently, a study with WNV-infected blood donors revealed that symptom development was positively correlated with early, potent IFN-*α* production [246]. However, this robust IFN-I response was associated with an inability to maintain continuing immunity during WNV infection, suggesting that an increased initial inflammation may negatively impact the adaptive-T cell responses.

#### 2.2.4. Yellow Fever Virus (YFV)

YFV, the causative agent of yellow fever, is endemic in the tropical regions of Africa and the Americas and is transmitted to humans and nonhuman primates by *Aedes* spp. mosquitoes, including *Aedes aegypti* [150]. Despite the presence of an effective vaccine YF17D [247], YFV infection has remained a public health concern in restricted parts of the world, with an incidence of 200,000 cases per year, leading to about 30,000 deaths [150]. In humans, YFV infection produces variable clinical manifestations, ranging from asymptomatic infection to mild febrile illness and to a possibly fatal disease characterized by severe hepatitis, renal failure, hemorrhage, and shock [248].

IFN-I induction has been shown in YF17D-vaccinated human subjects [249, 250] and combined IFN-I and -III are crucial for controlling YFV infection in mice [251]. Recently, it has been reported that cells infected with YFV stimulated pDCs to produce IFN-I in a TLR7- and cell contact-dependent manner. Cells producing immature particles as well as capsid-free viral RNA participated in pDC stimulation [252].

NS4B and NS5 of YFV have been reported as IFN-I antagonists. YFV NS4B interacts with STING, blocking IFN-I stimulation [253]. Moreover, YFV NS5 inhibits IFN-I signaling via binding and inhibition of STAT2 following IFN-I-induced phosphorylation of STAT1 and requires K6 ubiquitination [254]. This IFN-induced ubiquitination of YFV NS5 is absent in murine cells resulting in a lack of binding of YFV NS5 and human STAT2 in murine cells, highlighting the importance of YFV ubiquitination in determining the host cell range for YFV [255, 256].

Gene expression microarray analysis in PBMCs from YF17D-vaccinated humans revealed an immune profile related to antiviral IFN-I responses [249], suggesting that IFN-I may play a role in effective protection in vaccinated subjects. Moreover, human DCs infected with vaccine virus YF17D and a chimeric YF17D/DENV2 produced higher levels of IFN-*α* than those infected with DENV-2 [193], also indicating an induction of IFN-I production by YF17D vaccine virus. Recently, it has been reported that an inherited IFNAR1 deficiency resulted in life-threatening complications of vaccination with the YF vaccine in a previously healthy individual [257].

The dual role of IFNs in protection against and pathogenesis of viral infections was suggested using a gene overexpression screening approach in human cells [258]. In this study, several ISGs (ADAR, FAM46C, LY6E, and MCOLN2) were identified as inhibitors of YFV, WNV, HCV, HIV, chikungunya virus (CHIKV), and Venezuelan equine encephalitis virus (VEEV) replication. Conversely, several ISGs were found to enhance the replication of YFV, WNV, CHIKV, and VEEV, highlighting the complexity of the IFN-I system.

#### 2.2.5. Japanese Encephalitis Virus (JEV)

JEV is a mosquito-borne flavivirus causing severe neurologic disease, characterized by flaccid paralysis, meningitis, and encephalitis [150]. JEV is transmitted to humans by *Culex* spp. mosquitoes and is maintained in a zoonotic cycle involving pigs as the major reservoir and water birds as carriers [259]. JEV infections primarily occur in Asia, where 35,000-50,000 cases and 10,000-15,000 deaths are reported annually [150].

JEV infection of human microglial cells has been shown to induce an innate immune response characterized by the production of IFN-*β* via IRF-3 activation and phosphorylation. The overexpression of the ubiquitin ligase TRIM21, which interacts with IRF-3 negatively, regulated this innate immune response by targeting IRF-3-mediated IFN-*β* production [260]. Recently, neuronal transcriptomic responses to JEV infection showed the upregulation of RIG-I and MDA5, suggesting that neuronal cells play a significant role in immunity against JEV [261].

The antagonist activity of JEV NS5 in IFN-I responses has been reported. The JEV NS5 has been shown to block IFN-I signaling by reducing the phosphorylation of TYK2 and STAT1 and subsequently inhibiting STAT1 nuclear localization [262, 263]. Furthermore, JEV NS5 inhibited the nuclear translocation of IRF-3 and NF-*κ*B by binding to nuclear transport proteins KPNA3 and KPNA4, impairing the production of IFN-*β* [264].

The IFN-I response during JEV infection in humans has not been fully characterized. A study of proinflammatory profile in humans with JE revealed that nonsurviving patients with JE showed higher levels of IFN-*α* in cerebrospinal fluid than those from survivors during the first days of illness, suggesting that it may be associated with higher viral load [265].

#### 2.2.6. Saint Louis Encephalitis Virus (SLEV)

SLEV is a mosquito-borne flavivirus transmitted to humans by *Culex* spp. mosquitoes, first discovered in 1933 when a large epidemic of encephalitis occurred in St. Louis, Missouri [150]. SLEV distribution ranges from Canada to Argentina and across North America [266]. SLEV produces a mild febrile disease in children and young adults and severe neurologic manifestations that are more frequently observed in elderly and immunocompromised patients.

Information about IFN-I induction and signaling as well as IFN-I evasion by viral proteins after SLEV infection in human cells is currently very limited. Former studies have shown a variable effect of human IFN in primary human fetal glial cell cultures [267]. Similar to DENV, ZIKV, and WNV, the protective role of IFN-I against SLEV has been demonstrated in mice since IFN signaling-deficient mice are more susceptible to infection by SLEV than in immunocompetent mice [268]. Specifically, the protective effects of IFN-*α* have been shown in mice by reducing mortality from SLEV delivered by the aerosol and subcutaneous routes [269].

## 3. IFN-I Therapy and Pathogenic Effects

IFNs are increasingly recognized as therapeutic agents. Three different types of human IFN (*α*, *β*, and *γ*) are widely used for the treatment of various diseases due to their immunomodulating, antiviral, and antiproliferative properties [270, 271]. However, the optimal dose and duration of IFNs as therapeutic agents have not been established [272]. This is important since IFNs administered in pharmacological doses produce considerable toxicity that is dose-related and that may require cessation of therapy. Common side effects due to IFN-*α* include flu-like symptoms (fatigue, fever, myalgias, and headaches), pulmonary toxicity, gastrointestinal symptoms, neurotoxicity, and depression [273–275]. Lethal toxicity associated with IFN-*α* regimen is rare and severe toxicity due to IFN-*α* is manageable if recognized expeditiously [276, 277].

The role of endogenous and/or exogenous IFN in the viral infections discussed in this review has been partially explored in human cell lines, animal models, and clinical studies. Depending on the virus, the model used, and the time of infection, IFNs can be either beneficial or deleterious (Table 1).

Regarding influenza disease, a pathogenic role for endogenous IFN-I has been suggested in children with influenza infection, in whom clinical disease severity was associated with an increased level of IFN-*α* [278]. In agreement with this, another study associated severity with epithelial cell damage mediated by TNF-*α* related apoptosis-inducing ligand (TRAIL) [279] whose expression can be induced by IFN-*α* and IFN-*β* [280]. Moreover, recent evidence showed that the upregulation of TRAIL expression by monocytes and death receptor 5 (DR5) expression by epithelial cells contributed to the pathogenic mechanism induced by IFN-*α*/*β*, where enhanced production of this molecule can contribute to immunopathology in severe infections [64].

Concerning RSV infections, intranasal administration of recombinant IFN to infants with RSV infection has shown to be safe and to decrease the duration of symptoms without affecting the time of viral shedding [281–283].

During the 1980s, different studies showed that the use of high doses of intranasal IFN-*α* were useful in the prevention of HRV infection [284–286]. However, it was also found that the use of this molecule (in high doses) was associated with the occurrence of undesirable effects, as described above. Although low-dose treatment has proven to be better tolerated, it has been ineffective as postexposure prophylaxis [287]. Further investigation regarding the role of IFN-I during HRV infections needs to be completed.

Dual effects of IFN-I have been observed in HCoV infections, as mentioned previously. A series of case studies describing moderate and severe MERS-CoV infections in adults found that moderate cases had no IFN-*α* response, while severe cases had variable levels of IFN-*α* [288]. However, other studies have shown that MERS-CoV and SARS-CoV patients improved with IFN-*α* therapy [289, 290].

Regarding DENV infections, the studies evaluating the association of IFN-I response with disease severity in humans have yielded mixed results [188, 190–192], as described previously. Recent studies in dengue patients in South America have suggested that high levels of IFN- *β* might accompany a worsened progression of the disease [197, 198]. In addition, monocytes of individuals with past severe dengue (SD) exhibited a significant upregulation of IFNB-1, RIG-I, and NLRP3 genes compared to those with past non-SD that was accompanied with higher viral loads, suggesting that initial innate immune responses may influence disease outcome [291].

With regard to ZIKV, IFN-I treatment has not been described in ZIKV patients. A recent murine model of ZIKV infection using dexamethasone-immunosuppressed mice showed that IFN-I treatment improved clinical outcomes, reducing viral load and inflammation in different visceral organs including testicles, suggesting the consideration for evaluating the effects of recombinant IFN treatment in patients at high risk for ZIKV-associated complications [292].

Concerning WNV-infected patients, the use of IFN-*α* is limited to case reports of meningoencephalitis, where different outcomes have been described [293–296]. IFN-*α* treatment was well tolerated and might have potential beneficial effects. However, the dynamic course of WNV neuroinvasive disease prevented the determination of whether the beneficial effects were due to the experimental therapy or to chance. Further randomized, double-blinded, placebo-controlled clinical trials are needed to define the role of IFN-*α* treatment in WNV-infected patients.

The antiviral effects of IFN-*α* against JEV have been originally described in cell cultures [297]. In humans, IFN-*α*2a treatment against JE was also evaluated in a randomized double-blind placebo-controlled trial in Vietnamese children with JEV infection [298]. Intramuscular IFN-*α* administration did not show beneficial effects on hospital death or severe sequelae at discharge, suggesting that higher doses, alternative administration routes, or combination with other antiviral drugs might be needed.

Similar to WNV and JEV, the use of IFN-*α* was evaluated in human subjects with SLEV severe neurologic disease. A nonrandomized, unblinded, interventional pilot study evaluated IFN-*α*2b therapy for meningoencephalitis produced by SLEV and suggested a beneficial effect on the early neurologic course of the disease, indicating that two weeks of treatment were well tolerated [299]. A subsequent study in solid organ transplant recipients with SLEV meningoencephalitis showed that even with delayed administration, the combination treatment with IFN-*α*2b and intravenous immunoglobulin G was associated with a potential clinical improvement [300]. These studies support the conduction of a subsequent randomized, double-blinded, placebo-controlled trial of IFN-*α*2a therapy for SLEV meningoencephalitis.

## 4. Therapeutic Potential of ISG Effector Mechanisms in Human Viral Infectious Diseases

Upon recognition of viral infection, the cell, through PRRs, mediates the production of IFN-I leading to the transcription of hundreds of ISGs by the JAK/STAT pathway [301]. ISGs are the effectors of cell-autonomous antiviral defense and have been shown to be very effective at resisting and controlling pathogens. ISGs are induced to vastly different levels during viral infection or IFN treatment, and expression levels are often dependent on time, dose, and cell type. They act at different stages of the viral life cycle, from entry, replication, assembly, and release, providing adequate cellular immunity against both RNA and DNA viruses [302]. Microarray studies have identified between 50–1000 ISGs, with 200–500 genes typical of many cell types. Representative and well-studied ISG members with specific or broad antiviral activities include IRF1, IRF3, IRF7, IRF9, IFITM3, ISG15, and OASL. The products of these ISGs exert numerous antiviral effector functions, many of which are still not fully described. However, as the mechanisms of more IFN effectors are uncovered, it is likely that their modes of action will collectively span the majority of virus life cycle stages [303]. In addition to having potent antiviral activity, ISGs also augment the innate immune response to viral infection, thereby strengthening this response [304]. This has driven special attention in the attempt to advance novel therapeutics to control viral infection and their pathogenesis.

Currently, there are vaccines for influenza virus, YFV and JEV; however, neither effective vaccines nor specific therapies are available for RSV, hMPV, PIV, HRV, HCoV, DENV, ZIKV, WNV, or SLEV. Although several vaccines are under diverse phases of development for these viruses, there is a need for alternative antiviral therapeutic approaches.

Several ISGs have been described in the setting of respiratory viruses (Table 2). Recent work has shown that the egress of the influenza virus is also targeted by an ISG. Viperin interacts with the cellular enzyme farnesyl diphosphatesynthase to perturb lipid rafts, resulting in inhibition of influenza and rhinovirus release [305, 306]. In addition, ZAP, which can be upregulated independently from IFN production, limits influenza virus replication via the enhancement of RIG-I [307]. TRIM56 is another ISG with broad antiviral activity. Although its upregulation is dependent on IFN-I, it abrogates influenza virus A and B and HCoV infection through STING and TLR-3/TRIF [308, 309]. TRIM21 has shown activity against rhinovirus by intercepting incoming antibody-opsonized virions during cellular infection, mediating efficient postentry neutralization [310]. A new human IFN-induced gene that we have termed ISG20 codes for a 3′ to 5′ exonuclease with specificity for single-stranded RNA and, to a lesser extent, for DNA. ISG20-overexpressing HeLa cells showed resistance to infections by the influenza virus. The mechanism has been shown to be related to the impairment of the polymerase activity, inhibiting both the replication and transcription [311, 312]. Finally, TDRD7 (tudor domain containing 7) has shown activity against several paramyxoviruses, such as HPIV3 and RSV by interfering with the activation of AMP-dependent kinase (AMPK). The activation of AMPK is required for efficient replication. TDRD7 interferes with the activation of AMPK, limiting viral replication [313].

Several ISGs have been reported to inhibit flavivirus infection through different mechanisms (Table 2). The expression of IFN-I inducible ISGs such as IFITM2/3, viperin, ISG15, ISG20, OAS, BST2, RyDEN, TRIM69, and IFI6 has been shown to block DENV infection [314–320] at multiple steps of the viral cycle. IFITM2 and IFITM3 disrupted the early steps (entry and/or uncoating) of DENV and WNV infection [314, 316]. In contrast, three IFN-induced cellular enzymes, viperin, ISG20, and dsRNA-activated protein kinase, inhibited the steps of the DENV and WNV cycle in viral proteins and/or RNA biosynthesis [314]. DENV infection-induced viperin which has antiviral properties residing in the C-terminal region of the protein that acts to restrict early DENV RNA production/accumulation, potentially via the interaction of viperin with DENV NS3 and replication complexes [315]. The expression of the Repressor of yield of DENV (RyDEN) conferred resistance to all serotypes of DENV in human cells. RyDEN is likely to interfere with the translation of DENV via interaction with viral RNA and cellular mRNA-binding proteins, resulting in the inhibition of virus replication in infected cells [317]. OAS and its downstream effector RNase L have been reported to block DENV replication and likely contributed to host defense against DENV infection playing a role in determining the outcomes of DENV disease severity [318]. ZIKV has been reported to induce the expression of ISG15 in primary human corneal epithelial cells, and the silencing of ISG15 increased ZIKV infectivity [321]. ISG15 has also inhibited the replication of several flaviviruses, including DENV, WNV, and JEV [322–324]. IFN-*α*-inducible protein 6 (IFI6), an endoplasmic reticulum- (ER-) localized integral membrane effector, prophylactically protected uninfected cells by preventing the formation of virus-induced ER membrane invaginations that house flavivirus (YFV, WNV, and DENV) replication machinery [320]. TRIM69 interacts with DENV NS3 directly and mediates its ubiquitination and degradation, thus, interrupting DENV replication [319]. Recently, Schlafen 11, an ISG that controls the synthesis of proteins by regulating tRNA abundance, has been reported to restrict WNV, DENV, and ZIKV replication by impairing viral infectivity [325].

## 5. Conclusions

After 60 years of research, the protective role of IFN-I has been demonstrated from cell culture to animal models and in human subjects. More recently, pathogenic effects of IFN-I have been described during viral infections highlighting the vast and intricate interactions of IFN-I in the immune response. Further insights into the effector mechanisms of individual ISGs in the complex signaling networks in human viral diseases are needed in order to design more specific and effective therapeutic strategies.

## Figures and Tables

**Table 1:** Role of IFN-I in respiratory virus and flavivirus infections.

Virus	Model	Effect	Main finding	References
Human respiratory viruses				
Influenza virus	Human (*in vivo*)	Protective	Hospitalized subjects show enrichment for a minor *IFITM3* allele that leads to reduced influenza virus restriction	[70]
	Human (*in vivo*)	Pathogenic	High levels of IFN-*α*2 at enrollment predicted progression to severe disease	[278]

RSV	Human (*in vitro*)	Protective	Age and premature birth were independently associated with attenuated RIG-I-dependent IFN-*α* responses	[89]
	Human (*in vivo*)	Protective	Infants with severe RSV bronchiolitis have lower type-I IFN levels	[90]

hMPV	Mouse (*in vivo*)	Pathogenic	IFN-I contributed to disease pathogenesis due to increased inflammatory lung disease during infection	[102]

PIV	Human (*in vivo*)	Protective	Mean quantities of the virus in the secretions of those children with interferon was significantly lower compared to those without detectable IFN	[65, 117]

HRV	Human (*in vitro*) Human (*in vivo*)	Protective	Exogenous IFN-*α*, IFN-*β* significantly reduce HRV replication	[130, 131]

HCoV	Human (*in vivo*)	Pathogenic	High levels of IFN correlated with early sequelae	[148]
	Human (*in vivo*)	Protective	Patients treated with IFN show clinical response	[289, 290]

Emerging flaviviruses				
DENV	Human (*in vivo*)	Protective	Higher levels of IFN-*α* are observed in patients with milder dengue disease	[77, 190, 193–196]
	Human (*in vivo*)	Pathogenic	Higher levels of IFN-*β* in severe vs. nonsevere dengue children	[197]
	Human (*in vivo*)	Pathogenic	Higher levels of IFN-*β* in primary DHF patients compared to those with primary DF	[198]

ZIKV	Human (*in vivo*)	Protective	SNP at TLR-3 that decreased IFN-I response has been associated with microcephaly in newborns	[232]
	Human (*in vitro*)	Protective	Replication of Asian ZIKV strain Brazil 2015 (associated with neurodevelopmental disorders) was less sensitive to IFN-I, compared to other ZIKV strains	[233]

WNV	Human (*in vitro*)	Protective	PI3K that induces IFN-I controls WNV infection	[238]
	Human (*in vitro*)	Protective	Early activation of RLR or IFN-I signaling could block WNV infection	[239]
	Human (*in vivo*)	Inconclusive	IFN-*α* treatment was well tolerated and might have potential beneficial effects, due to treatment or chance	[293–296]

YFV	Human (*in vitro*)	Protective/pathogenic	ISGs shown to inhibit or enhance viral replication	[258]

JEV	Human (*in vitro*)	Protective	IFN-*α* at higher concentrations showed more efficacy in combating the replication of JEV	[297]
	Human (*in vivo*)	Inconclusive	No benefits from IFN-*α*2a treatment against children with JEV infection	[298]

SLEV	Human (*in vivo*)	Protective	IFN-*α*2b therapy for meningoencephalitis suggested a beneficial effect on the early neurologic course of the disease and clinical improvement	[299, 300]

**Table 2:** Some interferon-stimulated genes that inhibit respiratory virus and flavivirus infections.

ISG	Viruses shown to be susceptible	Mechanism/component of innate immune system augmented by ISG	References
	Respiratory viruses		
Viperin	Influenza A virus HRV RSV	TLR-7/9 (IRAK1/TRAF6) NF-*κ*B1/p50, AP-1	[305, 306]

ZAP	Influenza A virus	RIG-I	[307]

TRIM56	Influenza A and B viruses HCoV OC43	STING, TLR-3/TRIF	[308, 309]

TRIM21	HRV	cGAS, RIG-I,	[310]

ISG20	Influenza A and B viruses	Nucleoprotein blockade	[311, 312]

TDRD7	RSV PIV	Autophagy inhibition	[313]

	Flaviviruses		
IFITM2/3	DENV WNV	Entry and/or uncoating disruption	[314, 316]

Viperin	DENV	Restriction of early DENV RNA production/accumulation, via interaction with DENV NS3 and replication complexes	[315]

RyDEN	DENV	Interference with DENV translation via interaction with viral RNA and cellular mRNA-binding proteins	[317]

OAS	DENV	Blockade in DENV replication	[318]

ISG15	DENV ZIKV WNV JEV	Inhibition of viral replication	[321–324]

IFI6	YFV WNV DENV	Prevention of virus-induced ER membrane invaginations formation, that house replication machinery	[320]

TRIM69	DENV	DENV NS3 ubiquitination and degradation, thus interrupting DENV replication	[319]

Schlafen 11	WNV DENV ZIKV	Viral replication restriction by regulating tRNA abundance	[325]
